# On Analyzing Capnogram as a Novel Method for Screening COVID-19: A Review on Assessment Methods for COVID-19

**DOI:** 10.3390/life11101101

**Published:** 2021-10-17

**Authors:** M. B. Malarvili, Mushikiwabeza Alexie, Nadhira Dahari, Anhar Kamarudin

**Affiliations:** 1 School of Biomedical and Health Science Engineering, Universiti Teknologi Malaysia, Skudai, Johor Bahru 81310, Malaysia; alexie.uwabeza20@gmail.com (M.A.); nadhira@utm.my (N.D.); 2 College of Science and Technology (CST), Center or Excellence in Biomedical Engineering and E-Health (CEBE), University of Rwanda, KN 67 Street Nyarugenge, Kigali 3900, Rwanda; 3 Faculty of Medicine, University Malaya Medical Centre (UMMC), Kuala Lumpur 59100, Malaysia; anharkmy@um.edu.my

**Keywords:** COVID-19, diagnostic test, screening tool, SARS CoV-2 infection, CO_2_ waveform, feature extraction

## Abstract

In November 2019, the novel coronavirus disease COVID-19 was reported in Wuhan city, China, and was reported in other countries around the globe. COVID-19 is caused by severe acute respiratory syndrome coronavirus 2 (SARS-CoV-2) infection. Strategies such as contact tracing and a vaccination program have been imposed to keep COVID-19 under control. Furthermore, a fast, noninvasive and reliable testing device is needed urgently to detect COVID-19, so that contact can be isolated and ringfenced before the virus spreads. Although the reverse transcription polymerase chain reaction (RT-PCR) test is considered the gold standard method for the diagnosis of SARS-CoV-2 infection, this test presents some limitations which cause delays in detecting the disease. The antigen rapid test (ART) test, on the other hand, is faster and cheaper than PCR, but is less sensitive, and may limit SARS-CoV-2 detection. While other tests are being developed, accurate, noninvasive and easy-to-use testing tools are in high demand for the rapid and extensive diagnosis of the disease. Therefore, this paper reviews current diagnostic methods for COVID-19. Following this, we propose the use of expired carbon dioxide (CO_2_) as an early screening tool for SARS-CoV-2 infection. This system has already been developed and has been tested on asthmatic patients. It has been proven that expired CO_2_, also known as capnogram, can help differentiate between respiratory conditions and, therefore, could be used to detect SARS-CoV-2 infection, as it causes respiratory tract-related diseases.

## 1. Introduction

Coronavirus disease 2019 (COVID-19) is an infectious disease caused by a novel coronavirus known as SARS-CoV-2 [1]. Coronaviruses are members of the Coronaviridae family of the order of Nidovirales that mainly cause infections in the respiratory tract [2]. All viruses belonging to this order are enveloped, non-segmented positive-sense RNA viruses [3]. The novel coronavirus (SARS-CoV-2) was reported in Wuhan, the largest city of the Hubei Province in China, in November 2019 [4]. With the movement of population, SARS-CoV-2 infection was reported in China and many other countries around the globe. And the pandemic continues. On 30 January 2020, the World Health Organization (WHO) announced that the outbreak fulfils the criteria for a Public Health Emergency of International Concern [5]. The COVID-19 pandemic is taking a tremendous toll worldwide, mainly on families, societies, healthcare sectors, and on economies [6]. On 3 February 2021, the WHO report showed that the number of confirmed COVID-19 cases was 103,362,039 globally, including 2,244,713 deaths [7]. Malaysia had 226,912 confirmed cases and 809 deaths [8].

The primary route of SARS-CoV-2 infection is person-to-person transmission by direct contact. Otherwise, it can travel indirectly through respiratory droplets and fomites [4]. Therefore, affected countries have taken extensive measures to prevent and control the infection, including the detection of suspected cases at an early stage, the isolation of infected persons during treatment, and quarantine [1]. Moreover, citizens were encouraged to stay home, work from home [9], wash hands regularly, and maintain social distancing [10]. A health screening strategy is being used as a primary means of testing for SARS-CoV-2 infection. Here, infrared thermometers are used to detect core body temperature, primarily at the entrances of public buildings including schools, hospitals, shopping malls, airports, etc. [11]. Non-contact infrared thermometers have gained popularity in detecting fever since they are portable, easy to use, and cost-effective. However, their low sensitivity and accuracy may reduce the effectiveness of this measure.

To date, there is no specific treatment for COVID-19 pneumonia. Early diagnosis of SARS-COV-2 infection can help in providing effective treatment to the infected person and reduce further transmission of the virus. Recent published works show that the diagnosis of COVID-19 is mainly based on clinical symptoms, in addition to the use of real-time RT-PCR, antibody tests, chest computed tomography (CT) imaging, and chest x-ray images. Although real-time RT-PCR testing is the main approach used for diagnosing SARS-CoV-2 infection, the effectiveness of this test is based on numerous factors, such as the laboratory equipment, the skills of the technicians in performing the test and interpreting the results, and the long time required to generate the results [9]. These factors can lead to a delay in the detection of the virus at the early stage of infection. The combined use of real-time RT-PCR tests with either chest CT or serological tests may increase sensitivity in SARS-CoV-2 infection detection [12,13]. In some hospitals, diagnosis has only been based on clinical and CT findings due to the shortage of RT-PCR kits [14]. However, some patients presented a normal CT in the first two days after symptom presentation [14]. Therefore, the development of new tools can contribute to the timely and accurate detection of this infectious disease.

The major challenges in developing such a tool include identifying the best biosensor technology and the optimal parameters with sufficient sensitivity and specificity to assess respiratory function and its changes. Hence, a rigorous and extensive study was carried out from December 2019 to May 2021 through Google Scholar, Web of Science, PubMed, and Scopus, using different keywords (e.g., coronavirus, severe acute respiratory syndrome monitoring device, respiratory CO_2_ monitoring device, SARS-CoV-2 monitoring device, capnograph, COVID-19, capnogram) to identify appropriate respiratory disease assessment tools. We also manually searched the references of the selected articles for additional relevant material. In this paper, we reviewed assessment methods, which include the diagnostic tests, screening tools, and medical devices that have been used throughout the COVID-19 emergency. A complete detailed review is, however, beyond the scope of this paper, and can be found in the Joint Research Centre of the European Medicines Agency/European Commissions (https://covid-19-diagnostics.jrc.ec.europa.eu/devices, accessed on 1 June 2021). Based on our literature review, we propose using the features of CO_2_ signals as a screening tool for SARS-CoV-2 infection.

## 2. Biological Properties of SARS-CoV-2

SARS-CoV-2 is a large, spherical, enveloped, non-segmented, positive-sense, single-stranded RNA virus genome of about 30 kb that encodes for multiple structural and non-structural proteins. It consists of four main structural proteins, which are spike glycoprotein (S), membrane (M), envelope (E), and nucleocapsid (N) proteins. Neutralizing antibodies targeting a conserved region on the spike proteins of SARS-CoV-2 and SARS-CoV may be useful for treating COVID-19 [15]. These neutralizing antibodies are mainly involved in guiding the entry of viral particles into the host cells to infect them. S proteins contain S1 and S2 domains, and the interaction between the S1 domains of SARS-CoV-2 and a specific host cell receptor called Angiotensin Converting Enzyme 2 (ACE-2) promotes a conformational change in the S protein. The virus mediates membrane fusion with the host cell via the S2 domain and enters the host cell (specifically alveolar epithelial cells) [15]. The E protein plays a role in the production and maturation of the virus, the M protein determines the shape of the virus, and the N protein is involved in viral replication [16]. Coronavirus belongs to the family Coronaviridae and, to date, four coronavirus genera have been identified, including Alphacoronavirus, Betacoronavirus, Gammacoronavirus and Deltacoronavirus. These groups of viruses have been known to infect animals, including birds and mammals. SARS-CoV-2 is also a zoonotic coronavirus similar to SARS (severe acute respiratory syndrome) and MERS (Middle East respiratory syndrome) of the genus Betacoronavirus [17]. Tang et al. conducted a population genetic analysis of 103 SARS-CoV-2 genomes, and classified two prevalent evolutionary types of SARS-CoV-2, L type (~70%) and S type (~30%). The study showed that strains of the L type, derived from the S type, are evolutionarily more aggressive and contagious [18].

**Figure 1:**
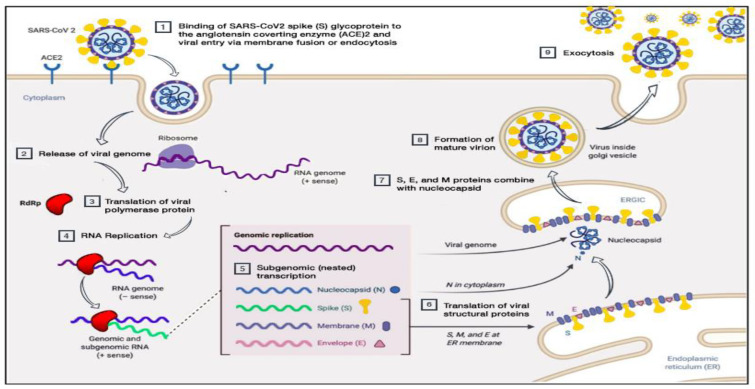
Pathogenesis of SARS-CoV-2 (adapted from Alanagreh, L. A., Alzoughool, F., and Atoum, M. (2020)). The human coronavirus disease COVID-19: its origin, characteristics, and insights into potential drugs and its mechanisms. *Pathogens*, *9*(5), 331) [19].

The SARS-CoV-2 virus may enter humans through the respiratory tract or conjunctival mucosa, and exhibits a preferential tropism for human airway epithelial cells. However, the pathological changes of the disease and its pathogenesis in humans are not clearly understood. The virus has a preferential tropism for human airway epithelial cells, and its cellular receptor, similar to SARS-CoV, is ACE2. The virion binds to the cellular receptor of angiotensin-converting enzyme 2 (ACE2) through a glycoprotein found on its surface (Figure 1). Following the virus’ entry into the host cell, the viral RNA enters the cytoplasm, where the structural proteins are located and the nucleocapsids are assembled. This is followed by the budding of the lumen of the endoplasmic reticulum and Golgi intermediate compartment, after which virions are released from the infected cell through exocytosis [19]. The expression of ACE2 is higher in minor salivary glands than in lungs, suggesting that salivary glands could be potential targets for COVID-19 detection. There are three different potential pathways of SARS-CoV-2 entry in the saliva (first, from the lower and upper respiratory tracts; second, from the blood into the gingival crevicular fluid; and third, by major and minor salivary gland infection) [20].

## 3. Clinical Manifestations of COVID-19

Despite the wide spread of SARS-CoV-2 infection, the disease’s clinical manifestations are nonspecific. At initial presentation, most COVID-19 patients manifest fever, a dry cough, and myalgia or fatigue [1,9,21]. However, some patients are asymptomatic [22]. Fever and cough are the predominant symptoms at the early stages of the illness, whereas diarrhea, a sore throat, and chest tightness are rare [4,23]. In the study carried out by [22] on 262 patients, 82.1% had fever, while 45.8% had a cough. This is consistent with a study by Chen, Jun, et al. [24], wherein 87.1% of the 249 patients presented fever, 36.5% had a cough, and only 3.2% had diarrhea [24]. Dyspnea is uncommon in COVID-19 patients, though it may be considered when classifying the severity of the disease [22]. Older males and patients with comorbidities such as hypertension, diabetes, and coronary heart disease are more vulnerable to SARS-CoV-2 infection due to their weaker immune systems [25,26].

During the disease’s course, some patients develop acute respiratory distress syndrome (ARDS) and septic shock, which leads to multiple-organ failure, including liver dysfunction, heart failure, and abnormalities in renal function associated with increased blood urea nitrogen [27]. Besides this, the patient may experience variations in their levels of blood elements, such as increased neutrophils, elevated C-reactive protein and reduced lymphocytes counts (particularly T-lymphocytes and hemoglobin). The study of Chen et al. [25] showed that out of 99 patients, 9% presented low leucocyte counts, 38% had high neutrophil counts, and 35% manifested lymphocyte counts below the normal range [25]. Reduced numbers of T-lymphocytes were also identified in patients with severe acute respiratory syndrome coronavirus infection (SARS-CoV), which emerged in November 2002. An absolute lymphocyte count below the normal range could be considered as a reference index in clinical settings while assessing novel coronavirus [25].

**Figure 2:**
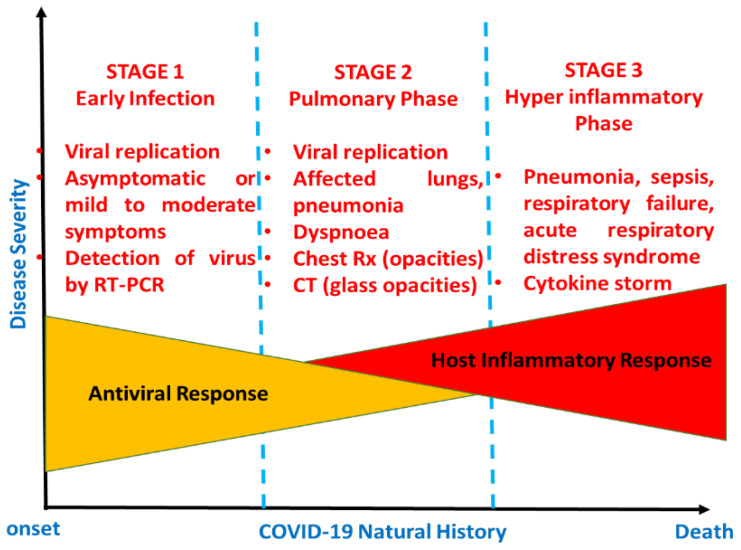
Schematic representation of the natural history of COVID-19.

COVID-19-infected patients are also more likely to experience blood coagulation disorders, especially those with cardiac injury [27]. In the study of Chen et al. [25], various blood coagulation tests were considered. Out of 99 COVID-19 patients,16% had an activated partial thromboplastin time (APTT) below the normal range, 30% had a prolonged prothrombin time, and 36% had D-dimer levels above the normal range [25]. A three-second longer prothrombin time and a five-second longer APTT were classified as a coagulopathy condition [26]. Wang et al. [28] reported on 138 cases with COVID-19, 58% of which had extended prothrombin times, and the levels of D-dimer were higher in ICU patients compared to non-ICU patients [28]. It was suggested that a level of D-dimer above 1 μg/mL would indicate poor prognosis at the onset of illness [28]. Venous thromboembolism is another complication faced by some hospitalized COVID-19 patients as a result of limited movement during illness, dehydration, or the presence of chronic underlying conditions such as hypertension, diabetes, or cardiac-related diseases [27]. Although SARS-CoV-2 has mainly been identified as a respiratory tract infection [29], it affects numerous systems, including the gastrointestinal, cardiovascular, respiratory, and immune systems. Therefore, both clinical symptoms and findings from diagnostic test such as PCR or imaging should be taken into account for the proper detection of COVID-19. Figure 2 is a schematic representation of COVID-19′s developmental stages.

In Figure 2, the natural developmental stages of COVID-19 are illustrated from the onset to recovery or death [30]. Normally, there are three stages, categorized via disease severity. Stage 1 is early infection, which is basically related to the onset of the disease and is generally characterized by the development of mild to moderate influenza-like symptoms, while the second phase is the pulmonary phase, wherein some individuals exhibit pneumonia-like symptoms. Lastly, stage 3 is characterized by hyperinflammation, wherein patients require admission to an intensive care unit (ICU) [30].

## 4. Diagnosis of COVID-19

Laboratory testing is important to confirming, isolating, and managing each case. It involves the detection and characterization of the etiological agent of SARS-CoV-2 in order to understand the disease’s epidemiology and management, and measures to suppress its transmission [31]. COVID-19 usually presents as an acute viral respiratory tract infection, and carries various common indications of viral pneumonia diseases such as influenza, parainfluenza, adenovirus infection, respiratory syncytial virus infection, metapneumovirus infection, and atypical pathogen infections such as mycoplasma pneumoniae and clamydophila pneumoniae. It is essential to trace travel and exposure history when approaching a suspected patient back from an epidemic area [32]. There are three main steps in testing:

1. Collection of samples (this involves the collection of samples at the right time and using the right technique);
2. Transportation of samples (this involves maintaining a cold chain and assessing the duration of transport);
3. Testing samples (this involves using the most suitable method for analysis).

Early detection is extremely useful in controlling the spread of SARS-CoV-2. Various studies reported the presence of SARS-CoV-2 infection in different clinical specimens, such as bronchoalveolar lavage fluid, sputum, saliva, throat cells, stool, nasopharyngeal (NPS) and oropharyngeal (OPS) swabs, blood, fibrobronchoscope brush biopsies, feces, and urine [28,33,34]. BLF had the highest positive results (93%), whereas pharyngeal swabs had the lowest (32%) among the lower respiratory track samples. Sputum and nasal swab samples exhibited 72% and 63% positive results, while fibrobronchoscope brush biopsies had a positive rate of 46%. The infection was found in feces at the rate of 29%. In the blood samples, the positive rate was only 1%. Of the 72 urine specimens sampled, all presented negative results [34]. SARS-CoV-2 infection is rarely present in the blood, and is absent in urine samples [29,34].

Bronchoalveolar lavage fluid (BLF) is collected for the diagnosis and detection of viral RNAs, particularly in severe cases, although a suction tool is required in this sampling process, and it is painful to the patient [35]. Nasopharyngeal (NP) and/or oropharyngeal (OP) swabs are often recommended for the screening or diagnosis of early infection, as recommended by the WHO. However, during the sampling process, healthcare workers are exposed to SARS-CoV-2 and other unknown pathogens via aerosols from swab [33]. In addition, NP swab specimens are obtained invasively by inserting the swab deeply into the nasal cavity. This can cause discomfort and minor injuries to the patients, such as bleeding in the mucosal layer [33]. Sputum and nasal swabs are mostly used for the diagnosis of SARS-CoV-2 infection, as their collection process is simple, fast and safe [35]. Although sputum has been identified as the most sensitive specimen for detecting the virus, a study conducted on 41 COVID-19 patients showed that only a small number of patients (28%) displayed sputum production as a symptom [36]. Therefore, nasal swabs seem to be the most commonly applicable specimens in the detection of SARS-CoV-2 infection [37].

## 5. Existing Diagnostic Tools

Currently, the clinical diagnosis of COVID-19 pneumonia is based on real-time RT-PCR tests, chest CT imaging, and the analysis of some hematology parameters [8] such as leukocyte or lymphocyte count [38]. In Wuhan, where the first cases were identified, diagnosis was firstly based on epidemiological factors, assessing whether the suspected patient had been in contact with wildlife, had been to Wuhan, or had a history of close contact with people from Wuhan or patients who had tested positive in the previous two weeks [1,4]. Thereafter, chest imaging and the detection of infection agents in respiratory, blood, and fecal samples were performed. Via the real-time RT-PCR method, SARS-CoV-2 infection was detected in lower respiratory tract samples [36].

### 5.1. Real-Time RT-PCR Test-Molecular Test

Real-time RT-PCR is a diagnostic test that relies on the nucleic acid amplification approach. The test is performed in vitro to detect the presence of viruses from sera and respiratory specimens, including nasopharyngeal swabs, lower respiratory tract aspirates, and sputum [39,40]. This test involves the use of reagents called “primers and probe”, as well as other important enzymes, which are used to magnify the target for detection. The SARS-CoV-2 genome encodes four structural proteins (i.e., the spike surface glycoprotein (S), nucleocapsid (N), membrane (M) protein, and the small envelope (E) protein) [41]. The N and E protein genes are the targets for amplification in the rRT-PCR assay, combined with open reading frame 1 (ORF1) ab and the RNA-dependent RNA polymerase (RdRP) gene. Real-time RT-PCR-based assays usually detect only two or three of these genes, which is sufficient to allow for rapid testing and diagnosis. However, interpreting the results may be challenging [41].

The real-time RT-PCR test remains the gold standard in diagnosing COVID-19. However, it presents some limitations, which are as follows. Firstly, the test must be performed in a certified and well-equipped laboratory by a well-trained professional capable of interpreting the results, and the generation of results takes a long time (2 to 3 h on average) [9]. Secondly, false-negative results may result from either the inappropriate collection, transportation, and handling of specimens, the presence of amplification inhibitors, or insufficient organism numbers in the specimen [40]. The results may also be affected by the quality of the RNA extracted from the swabs. The degradation of purified RNA, or the presence of RT-PCR inhibitors or genomic mutations may also cause false-negative results [42]. Moreover, real-time RT-PCR has a low detection rate at the initial presentation of the disease [3]. The identification of viral proteins using an antigen-based approach is a valid alternative for the rapid qualitative detection of SARS-CoV-2 infection [43].

### 5.2. Rapid Antigen Detection (RAD) Test

An antigen test is a qualitative method for detecting certain proteins that are present on or within a virus. Similar to a RT-PCR test, an antigen test also uses respiratory samples, including nasal and nasopharyngeal swabs [43]. Throat saliva and sputum are not commonly used for RAD tests [44]. Despite its low sensitivity, the antigen test is more cost-effective and faster than the real-time RT-PCR test. Different antigen test kits are being produced by manufacturers of diagnostic tests in different countries, and thereafter approved for emergency use. The Sofia 2 SARS Antigen Fluorescent Immunoassay (FIA) is a lateral flow immunofluorescent sandwich assay developed by Quidel Company, San Diego, USA and Coris BioConcept, Gembloux, Belgium. The Food and Drug Administration (FDA) issued an Emergency Use Authorization for this test, which detects antigen from the nucleocapsid protein of the SARS-CoV-2 virus. This test can assess a high number of individuals per day, as its results are generated within 15 min. However, the test needs to be performed in laboratories certified by the Clinical Laboratory Improvement Amendments (CLIA) or in a patient care setting with a CLIA Certificate of Waiver [43]. The Adeptrix Corporation developed a bead-assisted mass spectrometry (BAMS) antigen test. For this, Avacta Life Sciences Limited supplied Affimer^®^ reagents, which coat beads that bind the particles of the virus. Every bead is analyzed using mass spectrometry for the presence of the virus. The BAMS antigen test is cost-effective, and no special laboratory equipment is required. Moreover, the test has a greater capacity, as numerous samples can be taken and analyzed by a single laboratory technician every day [45,46].

The COVID-19 Ag Respi-Strip is a type of RAD test developed by Coris BioConcept, Gembloux, Belgium. This test was authorized by the Belgian Federal Agency for Medicines and Health Products for use in public health institutes in Belgium [47]. This diagnostic method uses patient nasopharyngeal secretions, and the results are generated within 15 min. Despite its low sensitivity, which also depends on the type of specimen and the level of the viral load [47], the COVID-19 Ag Respi-Strip test is the first-line method of diagnosing COVID-19 in Belgium. RAD tests are inexpensive and easy to operate. However, their analytical performance is affected by a variety of factors, such as the viral load, the quality of the samples, and the samples processing method [48]. Rapid antigen tests are not recommended for use as standalone diagnostic tools in clinical practice due to their low sensitivity, which can give false-negative results [49].

### 5.3. Antibodies (Serology) Test

The antibody test, also known as the serology test, is a screening method that uses blood samples taken via finger prick or from a vein in the arm [49]. This test determines whether antibodies have been developed against the virus [43]. Antibodies are critical proteins to fighting and clearing out the virus, and they are produced by the immune system. When an infection is present in the body, adaptive immunity is expected to increase. B lymphocytes produce specific antibodies and CD8+ T cytotoxic lymphocytes that help eliminate infected cells [13]. COVID-19 patients develop antibodies against the nucleoprotein and receptor binding domain (RBD) of SARS-CoV-2. However, the window of antibody response varies depending on the type of antibody [50]. Zhao et al. [51] evaluated the dynamics of three different antibodies (total antibody (Ab), immunoglobin M (IgM) and immunoglobin G (IgG)) in relation to disease progression in COVID-19 patients [51]. The RNA test showed greatest sensitivity in the first week of illness. However, its sensitivity decreased in the later phases. In the last two phases after onset (weeks 8–14 and 15–39), the total Ab test presented its highest sensitivity (90% and 100%, respectively), and the IgM test had greater sensitivity than the IgG test from day 1 after onset to the last day (day 39) [51]. The Ab and IgG tests could thus help identify the level of humoral immunity in COVID-19 patients [51].

A combination of IgM and IgG antibodies provided increased sensitivity compared to either alone (IgM or IgG) [49]. Of 397 confirmed COVID-19 cases, 64.48% developed both IgM and IgG antibodies, whereas the number of patients that tested positive for only IgM antibodies was greater than those testing positive for IgG antibodies (18.13% and 6.04%, respectively) [49]. In the work of Guo et al. [52], Ig A antibodies were also assessed in 208 plasma samples collected from 82 confirmed COVID-19 cases and 58 probable cases [52]. In this study, probable cases were patients who had negative quantitative polymerase chain reaction (qPCR) test results but who presented typical clinical manifestations [52]. Almost all the samples were positive for IgA antibodies (93.3%), while IgM and IgG antibodies were present in 90.4 and 77.9%, respectively. IgA and IgM antibodies were both detected within a median of 5 days, whereas IgG was detected in 14 days [52]. Various studies reported the potential applicability of serology testing in the diagnosis of SARS-CoV-2 infection at different stages of illness. However, seroconversion is not the same in all individuals, and depends on the time taken for symptoms to manifest and the time at which the specimen was taken [52]. In addition, false-negatives result from low concentrations of antibodies [49]. Thus, antibody tests are not used as stand-alone diagnostic tests, and are not recommended in any setting wherein reliable diagnostics are crucial to avoiding the spread of the virus [53]. Antibody tests paired with RNA-based tests display enhanced sensitivity in detecting the novel coronavirus [51].

### 5.4. Chest Computed Tomography (CT)

Many researchers have highlighted the applicability of chest CT in COVID-19 diagnosis and evaluation, based on different imaging features such as ground glass opacity (GGO), consolidation, crazy paving patterns, the presence of a halo sign, and changes in the airways [1,54]. Ground glass opacity is defined as hazy opacity with bronchial and pulmonary vessel markings, whereas consolidation is a pathological process in which air that is normally present in the alveoli is replaced with fluids, blood or cells. It is characterized by increased pulmonary parenchymal density, which causes obscuration in the vessels and airway wall margins [54]. In [28], ground glass opacity was the most common feature in all patients. This agrees with Li et al. [55], who considered both clinical and CT findings in 83 COVID-19 cases. Of the 83 patients, 30.1% had a severe/critical illness, and 69.9% were non-severe. Ground glass opacity was common in all severe cases, while consolidation, bronchial wall thickening, and crazy paving patterns were present in 88, 64 and 56% of severe cases, respectively [55]. The CT finding of bronchial wall thickening marked changes in the airways, as did consolidation, interlobular septal thickening, crazy paving patterns, spider web signs, subpleural lines, etc. [56]. The number of lobes affected, the level of harm due to ground glass opacity and consolidation, the presence of nodules in the lungs, pleural effusion, and the distributions of opacities and patterns were also assessed in COVID-19 patients [57]. All 21 patients were free of pulmonary nodules and pleural effusion, 6 (28%) manifested both ground glass opacity and consolidation, while 4 (19%) presented crazy paving patterns. In three (14%) patients, the initial chest CT findings were normal, although their PCR test showed that all were positive for SARS-CoV-2 infection [57]. The arising of negative imaging results from confirmed COVID-19 patients shows that chest CT has limited sensitivity and reliability in detecting infection, especially at the onset of illness [57]. A combination of chest CT and real-time RT-PCR testing thus achieves accurate results in the early diagnosis of COVID-19 [12,58].

## 6. Current Screening Tools for COVID-19

### 6.1. Thermometers

At present, infrared thermometers are used to test for fever as a primary means of detecting SARS-CoV-2 infection. Measurement of core body temperature has become a requirement before entering public buildings, such as a shopping complexes, clinics, schools, airports, etc. [11]. This method was also used to detect SARS infection [59], which has similar clinical symptoms to SARS-CoV-2, including fever, cough and fatigue [60]. Most COVID-19 patients present fever at the onset of illness, and so screening body temperature is crucial to the rapid detection of suspected cases [61]. Non-contact infrared thermometers have gained popularity in detecting fever as they are portable, easy to use, do not cause discomfort, and do not depend on direct contact between the device and the forehead of the subject [62]. Although the device is not expensive and no constant recalibration is required, its sensitivity and accuracy are low compared to oral thermometers. This low accuracy can result from the distance between the operator and the subject, which is often greater than is recommended (3–15 cm) [62]. On the other hand, ear infrared thermometers have shown high accuracy in measuring body temperature, but they require direct contact with the subject, and so the probe must be frequently replaced to avoid the spread of the disease [63].

### 6.2. Thermal Imaging Systems

Thermal imaging cameras are another alternative non-contact tool for screening fever. As the subjects pass into the field of view of the camera, their thermal images are captured and analyzed [63]. The appropriate use of thermal imaging systems provides an accurate measurement of the surface skin temperature of an individual. However, this accuracy is affected by various factors, such as the setup of the system, the environment of the system, the skill of the operator, and the preparedness of the person who is being screened [64]. As SARS-CoV-2 infection continues to spread, a variety of temperature screening tools have been developed as a means of quickly and easily identifying suspected cases. Rokid Company, China, have developed T1 thermal glasses that can simultaneously screen the temperatures of up to 200 people within two minutes. These smart glasses are equipped with an infrared sensor, a Qualcomm CPU, and a 12-megapixel camera. The glasses can record both live photos and videos, and can detect the temperature of a person three meters away. These thermal imaging detection tools are being used in China by national authorities, national park staff, and schools [65]. Forward-looking infrared (FLIR) systems have also been introduced, in the form of two configurations of smart camera, namely, the A400/A700 Thermal Smart Sensor and the Thermal Image Streaming fixed camera. The high-quality Configurable Thermal Smart Sensor can detect elevated skin temperature in the targeted area, such as the forehead or the corner of the eye. Further screening is recommended for individuals with above-average skin temperature. FLIR thermal cameras are being used at airports to detect body the temperatures of passengers and flights crews [66]. Compared to non-contact infrared thermometers, thermal imaging systems have demonstrated an increased screening capacity. However, their precision in screening fever is lower, and they require regular calibration, as well as having a high initial cost [63]. Nonetheless, we cannot deny the usefulness of these systems for the initial detection of body temperature. However, they should not be used as definitive diagnostic tools of the presence of SARS-CoV-2, as some individuals may have COVID-19 but no fever [64].

## 7. Expired Carbon Dioxide Measurement: A New Screening Tool for COVID-19

### 7.1. CO_2_ Removal from Human Body

Human cells require oxygen (O_2_) and nutrients for their metabolism. The main byproduct of cell metabolism is CO_2_. CO_2_ is produced in all cells of the body, and we mainly depend on the lungs to remove it. Therefore, any problems with the lungs would be reflected in changes in CO_2_ levels in the blood [67]. The body compensates for any problems in the lungs by increasing breathing rate and excretion via the kidneys. These aspects can be monitored via blood samples. The CO_2_ levels in the blood can be measured in arterial blood samples and are usually expressed as the partial pressure of CO_2_ in mmHg or kPa. Expired CO_2_ concentrations can also be measured noninvasively via exhaled breath. A capnograph is a noninvasive device that uses infrared technology to measure the CO_2_ in expired gases and generates a continuous plot of exhaled CO_2_ over time, known as a capnogram. The use of infrared rays depends on the interaction between the CO_2_ molecules in the air and the infrared ray, which is emitted at a particular wavelength. The amount of light absorbed is directly proportional to the concentration of absorbing molecules—in this case, CO_2_.

**Figure 3:**
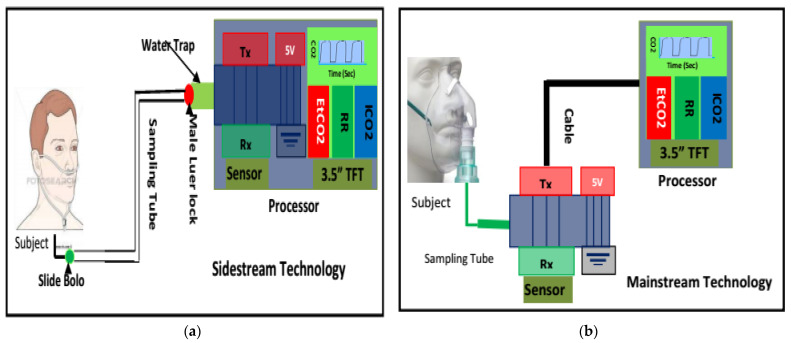
Capnography development techniques: (**a**) sidestream method incorporating a sample tube, scavenge, water trap, water-permeable tube, CO_2_ sensor, pump, processor and monitoring unit; (**b**) the main-stream method incorporates a processor and monitoring unit.

When developing a capnograph, the CO_2_ in expired breath is usually measured in one of two ways. First, mainstream capnography measures the CO_2_ flowing through an endotracheal tube (Figure 3b). 

This is the most accurate method of measuring CO_2_ and devising the expiration capnogram, because the flow of air within an endotracheal tube is laminar in nature [68]. Furthermore, the measurement can be taken without interfering with the flow of air itself. However, this method is primarily suitable for intubated patients, i.e., critically ill ventilated patients in intensive care units or in operating theatres. The American Society of Anesthesiologists (ASA) lists capnography as a standard monitoring parameter for all critically ill ventilated patients, noting that over 90% of adverse events are preventable via pulse oximetry and capnography monitoring alone [69].

**Figure 4:**
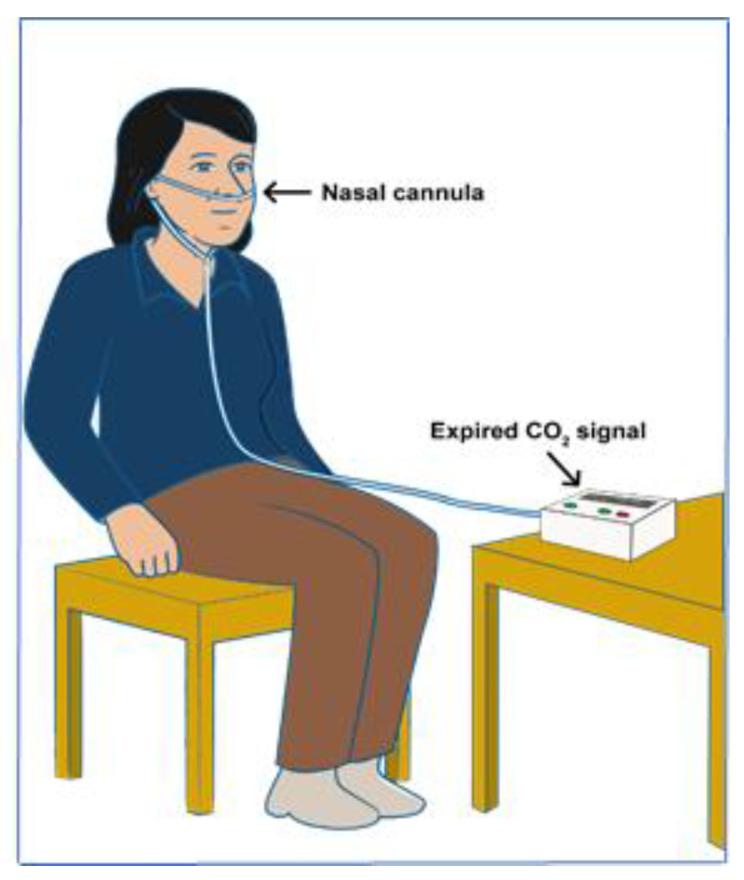
A subject during the recording of CO_2_ via the nasal cannula.

Secondly, sidestream capnography samples a small amount of air aspirated from the main exhaled stream, which is taken as close as possible to the nose or mouth to ensure a mostly laminar flow (Figure 3a). The main advantage of sidestream capnography is its ability to measure the CO_2_ levels in exhaled breath without interfering significantly with the patient’s breathing. This allows for the monitoring of spontaneously breathing nonintubated subjects, as samples can be obtained via nasal cannulas. Furthermore, the administration of oxygen can continue unimpeded through the nasal cannula [70,71,72]. In this regard, the use of sidestream monitoring is increasing in popularity due to the improvements in patient comfort and acceptance associated with it. Figure 3 illustrates both the sidestream and mainstream methods of capnography for measuring CO_2_. Figure 4 shows a typical capnogram waveform recording setup, wherein a nasal cannula is attached to the subject’s nose while they breathe at their own pace.

Today, capnography has been incorporated into intensive care units around the world, and routinely identifies issues in ventilated patients or those under anesthesia. For example, capnography waveforms and trends help identify overly rapid or inadequate breathing rates; blocked or obstructed breathing tubes; inappropriate ventilator settings; or when patients may be waking up from sedation and paralysis [73,74,75].

### 7.2. Interpretation of Capnogram

**Figure 5:**
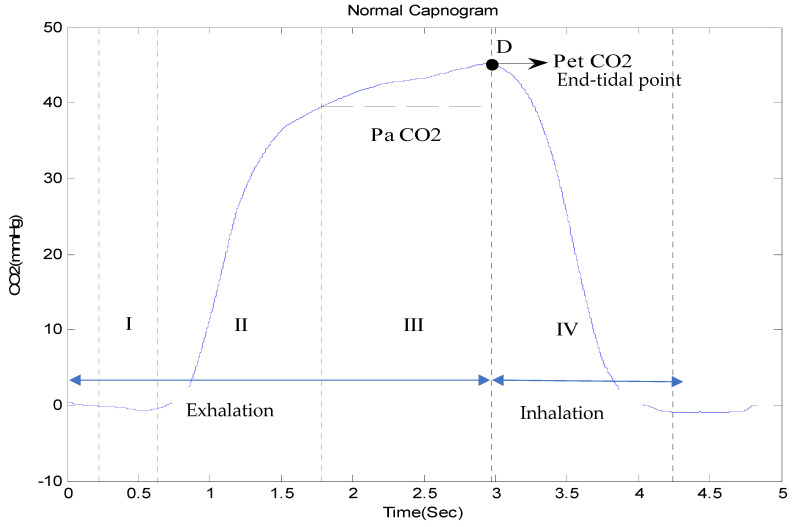
A complete breath cycle in a normal capnogram, with its four phases.

A capnogram illustrates variations in the partial pressure of CO_2_ expired during respiration [76]. A time-based capnogram is an instantaneous graphical display of the CO_2_ concentration (mmHg) versus time (seconds). It reflects development of the respiratory condition of a patient. A normal capnogram has four phases and an end-tidal point (Figure 5). Each phase reflects a section of the usual process of CO_2_ elimination [77]. The flat first phase represents relatively CO_2_-free early exhalation. As exhalation continues, the expired CO_2_ increases very rapidly, and this creates the near-vertical rise in phase II. Phase III begins near the end of the normal exhalation. The end of this plateau phase is marked D (the point at which the measured alveolar CO_2_ levels best approximate the partial pressure of CO_2_ in the arteries (PaCO_2_)). At this point, the level of sampled CO_2_ is referred to as either PetCO_2_ or etCO_2_ (the end-tidal point). As inspiration begins, the near-vertical, rapidly falling phase IV can be observed. When ventilation and perfusion are normal, PetCO_2_ should be 2–5 mmHg higher than PaCO_2_. The alveolar air is measured at the end of the horizontal plateau, or the end-tidal point (EtCO_2_), corresponding to the end of exhalation. This is usually the point with the highest CO_2_ reading. In normal lungs, etCO_2_ values are very close to blood CO_2_ values, with the former usually being just a few mmHg lower, as mentioned earlier.

**Figure 6:**
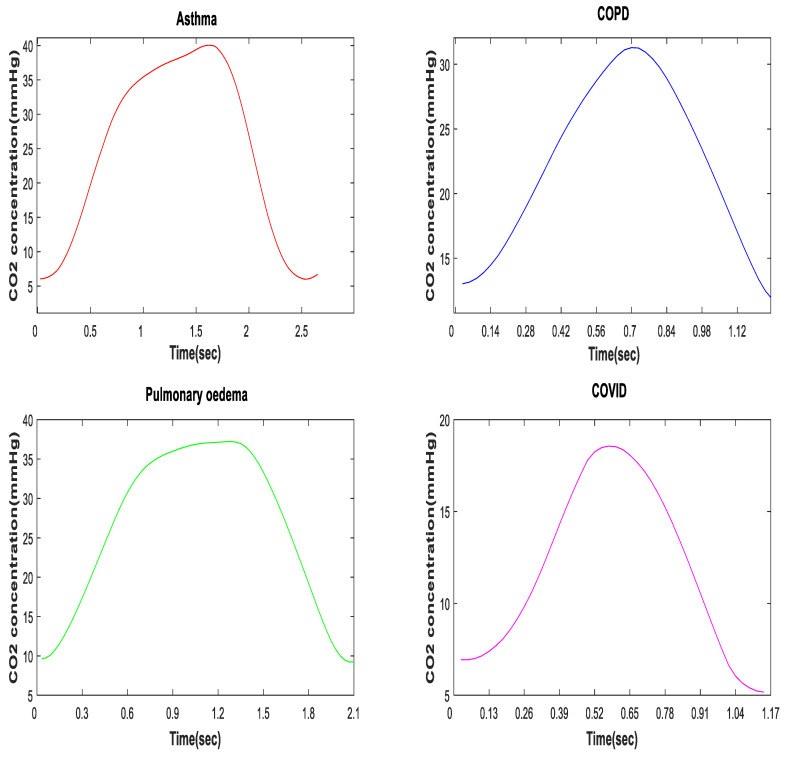
Morphology of a CO_2_ waveform in asthmatic, COPD, pulmonary edema, and SARS-CoV-2 patients.

In a healthy subject, the CO_2_ waveform, or the capnogram, has a square shape. This is comprised primarily of a rapid upstroke (indicating the “wall of alveoli air”, containing CO_2_, being discharged from normal lungs), a horizontal plateau (indicating the constant level of CO_2_ expelled from the alveoli) and a rapid downstroke (indicating the smooth flow of inspired air). However, the morphology of the capnogram changes due to changes in breathing, ventilation, airway obstructions, or other breathlessness-associated conditions. Figure 6 shows the morphological changes of a capnogram associated with various respiratory conditions, such as asthma, chronic obstructive pulmonary disease (COPD), pulmonary edema and SARS-CoV-2. The changes in an asthmatic capnogram primarily reflect the variations in the emptying of alveoli that are seen in asthma. Similar changes occur in other conditions of minor airway narrowing, such as COPD [78,79]. A complete overview of interpretations of capnograms for different conditions is, however, beyond the scope of this paper, but can be found elsewhere [70]. Smalhout, considered by many the father of clinical capnography, has published around 6000 capnograms, known as the atlas of strip-chart capnograms, which addresses numerous applications of capnography [80].

While some changes in the morphology of the CO_2_ waveform can be seen with the naked eye, such as the “shark fin” shape for asthmatic patients, more subtle variations require computation and pattern recognition methods. A deeper understanding of the shapes of COVID-19 capnograms, instead of just their etCO_2_ readings, can help in the development of a more effective screening tool for SARS-CoV-2 infection.

### 7.3. Analysis of Capnogram Waveform

Expired CO_2_ provides information that can assist physicians in identifying spot ventilation derangements, extubation outcomes, bronchospasms, and the effectiveness of therapy in the clinical environment [81]. Furthermore, features extracted from CO_2_ signals, such as end-tidal CO_2_, respiratory rate (RR), time spent at EtCO_2_, exhalation duration, Hjorth parameters (activity, mobility and complexity), the slope of phase II, end-exhalation slope, the slope ratio (SR), and the area ratio can be used to monitor and diagnose cardiopulmonary diseases, such as COPD, asthma, congestive heart failure (CHF), pulmonary embolism, and pneumonia [82,83].

Different researchers have reported on various time and frequency features of capnograms, including slopes, angles, Hjorth parameters (activity, mobility, and complexity), curvature measures localized around the transition from the ascending phase to the alveolar plateau, EtCO_2_, exhalation duration, time spent at EtCO_2_, power spectral density (PSD), energy, variance, skewness, and kurtosis [84,85]. These features have been used to quantify differences between the shape of a capnogram in a normal subject and that of a patient with an obstructive or restrictive disease Table 1. Capnogram features related to asthma have been widely explored, and have been correlated with spirometric indexes for discriminating asthmatic from non-asthmatic subjects and estimating asthma severity [86].

**Figure 7:**
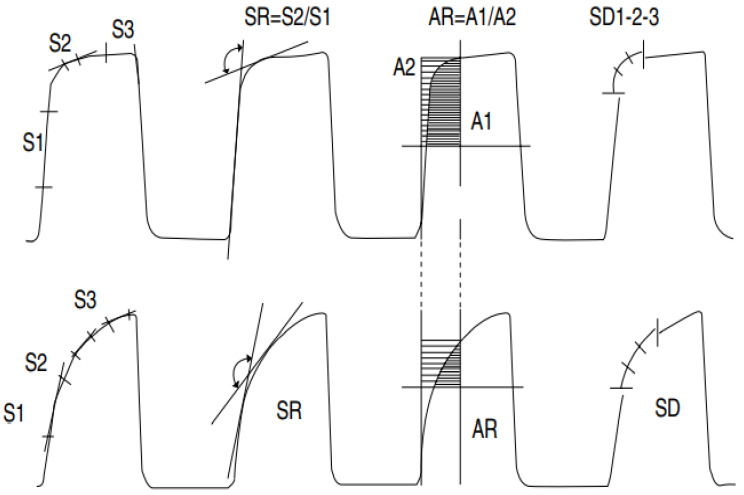
Illustration of capnogram and its eight associated indices: slopes (S1, S2, and S3), slope ratio (SR), areas (A1 and A2), and indices of the second derivative (SD1, SD2, and SD3). The upper capnogram shows the CO_2_ waveform of a normal individual, and the lower capnogram shows a deformed CO_2_ signal due to obstruction [84].

You et al. [84] evaluated the consecutive phases of capnograms for 30 asthmatic and 10 nonasthmatic subjects. In this study, eight capnographic indices (slopes S1, S2 and S3′s slope ratio (SR); area ratio (AR); SD1, SD2, SD3 (derivatives)) were analyzed (Figure 7). All these parameters were correlated with spirometric parameters, but the strongest correlation was observed by analyzing the angle between the ascending phase (E2) and the alveolar plateau (E3). The correlation between these indices shows that the severity of bronchospasm can be evaluated by quantitatively analyzing the shape of the capnogram [84]. That said, these capnographic indices were computed manually.

**Figure 8:**
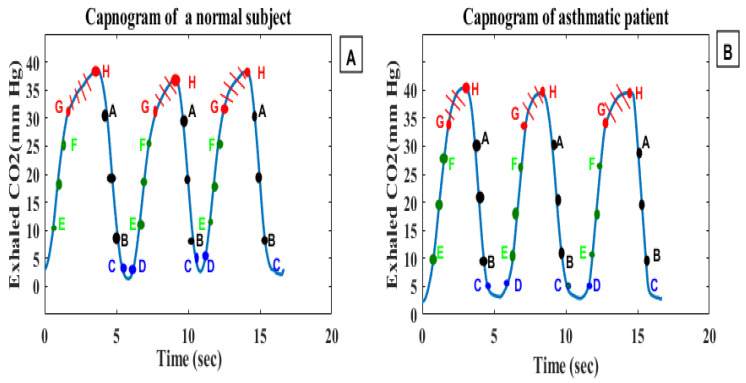
(**A**) represents a capnogram of a normal subject, (**B**) represents a capnogram of a subject with asthma; the three segments of interest are A–B, E–F, and G–H.

Yaron et al. [92] conducted a prospective study on 20 asthmatic patients and 28 healthy subjects to determine whether the alveolar plateau (dCO_2_/dt) can help detect bronchospasm in adult asthmatic patients, and the correlation between dCO_2_/dt and PEFR. In each patient, the dCO_2_/dt values of five consecutive regular breath cycles were measured manually, and the mean slope was calculated. The computed capnogram index (dCO_2_/dt) was correlated with the log of the predicted percent PEFR (r = 0.84, *p* < 0.001). Betancourt et al. [86] evaluated degrees of asthma severity using capnogram features obtained via the decomposition of a breath cycle into small segments (A–B, C–D, E–F and G–H), and further intermediate parts between the segments (B–C, D–E, F–G and H–A) (Figure 8). A support vector machine was used to classify asthma severity into six classes using feature vectors. The G–H segment presented the best results, with a sensitivity of 55.71%, specificity of 99.38%, correct error of 86.0%, and error rate of 13.91%. The results show that the terminal indices of the capnogram are highly sensitive to airway obstruction [86].

**Figure 9:**
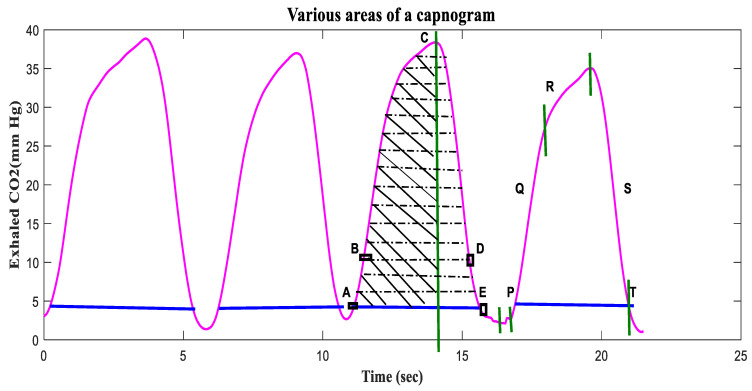
Various areas extracted from the second breath cycle of the recorded CO_2_ waveform: AR1 (A-B), AR2 (D-E), AR3 (A-B-C), and AR4 (A-B-C-D-E) [91].

Doğan, Nurettin Özgür, et al. [89] analyzed the variation in EtCO_2_ levels in COPD exacerbations. A total of 102 COPD patients (69 admitted and 33 discharged) were enrolled in the study. Their EtCO_2_ values were measured before and after treatment (Pre-EtCO_2_ and Post-EtCO_2_) and correlated with arterial partial carbon dioxide pressure levels (PCO_2_). The Pre-EtCO_2_ and Post-EtCO_2_ measurements correlated positively with PCO_2_ (r = 0.756, *p* < 0.001 and r = 0.629, *p* < 0.001). The median value of EtCO_2_ level before treatment was 39 mmHg in the admitted patients and 32 mmHg in the discharged patients. After treatment, the median value of EtCO_2_ level was reduced to 36 mmHg in the admitted patients, while that of discharged patients remained constant (32 mmHg). At 34.5 mmHg, the sensitivity and specificity of using the EtCO_2_ value to predict admission status were 65.2% and 63.6%, respectively. The authors concluded that EtCO_2_ levels provide little useful information for evaluating patients with exacerbated COPD in the emergency department [89]. Singh, O. P. et al. [91] computed various capnogram features, including the areas (ARi) AR1(A-B), AR2(D-E), AR3(A-B-C), and AR4(A-B-C-D-E), the sum (AR1 + AR2), and dCO_2_/dt, the derivative of the complete expiratory portion (A-C) (Figure 9). The areas AR1, AR2, AR3 and AR4 represent the upward expiration, downward inspiration, absolute exhalation and a complete breath cycle, respectively. The areas (AR1, AR2, AR3, AR4, and AR1 + AR2) for the asthmatic CO_2_ signal possess a higher mean value than the non-asthmatic CO_2_ signal, while the dCO_2_/dt of the expired phase decreased in the asthmatic patients compared to the nonasthmatic patients. These features may increase or decrease during an asthma attack, thus providing useful information related to asthmatic changes [91].

Modifications in the shape of the capnogram waveform have been analyzed by many researchers, and various features have been proposed as indicators of respiratory disorders, with asthma most common among these [84,86,91]. Moreover, Mieloszyk et al. [83] quantitatively analyzed capnogram waveforms to discriminate between COPD and congestive heart failure (CHF), and between COPD and normal patients. The modifications in the CO_2_ signal due to obstructive diseases are still a point of concern. In different studies, the slopes in different parts of a capnogram were measured, and were shown to be useful in detecting changes in the airways related to treatment [83,87]. In the study of Hisamuddin et al., capnographic waveform indices (slope of phase 2, slope of alveolar plateau and angle α) were analyzed post- and pre-treatment [87]. These features were found to be useful in detecting improvements in bronchospasm post-treatment. Howe et al. verified these three features (slope of phase 2, slope of alveolar plateau and angle α). The slopes were measured using linear trendline analysis, and the angle α was calculated from the observed gradient of phase 2 and phase 3. Before treatment, the mean gradient values of phase 2, phase 3 and angle α were 2.61, 0.44 and 134.36, respectively, whereas the post-treatment values were 2.74, 0.23 and 123.27, respectively. A minor change was noticed in the slope of phase 2 (*p* = 0.35), while the slope of phase 3 and angle α changed significantly, with *p* < 0.001 for both. The insignificant change observed in the slope of phase 2 might be due to the mistake made while selecting the starting point of phase 2 (at a CO_2_ value equal to 4 mmHg) [82].

Over the past decades, many studies have been conducted for the extraction of capnogram features in association with different respiratory conditions. Incorporation, implementation, and feasibility of these features in developing a real-time CO_2_ measurement system has been verified by Singh, O. P. et al. in [93] and Asher, R.J. et. al in [94]. The system in [93] has already been tested on asthmatic patients and available commercially as ashthma monitoring system. It has been proven that expired CO_2_, also known as capnogram, can differentiate various respiratory conditions and, therefore, we propose this study to investigate feasibility of expired CO_2_ to be used to detect SARS-CoV-2 infection, as it causes respiratory tract-related diseases.

### 7.4. Relationship of CO_2_ and SARS-CoV-2 Infection

The lung and airways are mainly affected by SARS-CoV-2 infection. An autopsy study of COVID-19 patients reported different pathological lesions, such as alveolar exudative inflammation and interstitial inflammation, alveolar epithelium proliferation, and hyaline membrane formation, in the lungs of those patients [95]. COVID-19 patients may also experience a lung injury that can lead to acute respiratory distress syndrome (ARDS) [96]. ARDS leads to respiratory failure resulting from the improper oxygenation and excretion of CO_2_. Patients with ARDS are also at risk of developing arterial hypoxemia due to ventilation-to-perfusion mismatch [97]. Besides this, minute ventilation and pulmonary dead space both increase a s result of the impaired elimination of CO_2_ from the body, which causes hypercapnia [98]. Hypercapnia is often caused by the failure to remove excess CO_2_, and it has been marked as a predictor of poor prognosis for COVID-19 patients [99].

The measurement of CO_2_ level is essential in airway management and the early detection of respiratory depression. The normal value of blood CO_2_ level ranges between 23 and 31 mmol/L [100,101]. A CO_2_ level ≤ 23 mmol/L has been considered to represent a decrease in COVID-19 patients [99]. Hu, Di et al. [99] evaluated the changes in CO_2_ level in 1776 COVID-19 patients with underlying diseases of different systems, including the cardiovascular, pulmonary, endocrine, neurology and digestive systems. Based on the level of CO_2_ in their blood, the patients were classified as either non-declined CO_2_ or declined CO_2_ patients; 75.6% of these patients showed a CO_2_ level in the normal range, while 24.3% had decreased CO_2_ levels. The majority of these patients had pulmonary diseases [99]. Reduced CO_2_ levels can result from shortness of breath, reductions in pulmonary perfusion, increased alveolar dead space, and hyperventilation. In [99], decreased CO_2_ levels were associated with a high mortality risk in COVID-19 patients, but were found to have no significant impact on the severity of disease [99]. The level of CO_2_ in the blood can be measured using a simple test of blood from an artery or vein [101]. However, this method involves arterial or venous puncture [100], which creates discomfort for the patient.

Unlike the CO_2_ blood test, capnography is an invasive method of continuously monitoring the CO_2_ exhaled in respiratory gases [101], and it could be effective in monitoring respiratory conditions in COVID-19 patients. In addition, quantitative analyses of capnogram shape (CO_2_ waveform), undertaken by extracting different features (such as slope ratio, time spent at EtCO_2_, exhalation duration, Hjorth parameters (activity, mobility and complexity), slopes in different regions of the CO_2_ curve, the slope ratio (SR), and the area ratio), can reveal real-time changes in respiratory systems, and can also help classify disease severity in COVID-19 patients. Based on this, we propose the use of CO_2_ signal features as a screening tool for SARS-CoV-2 infection, as reported below.

### 7.5. On the Capnogram as Feature for COVID-19 Detection

#### 7.5.1. Study Setting

The study was performed in the Emergency Department of the University Malaya Medical Centre, Kuala Lumpur, Malaysia. The study was approved by the Medical Research and Ethics Committee (MREC), Ministry of Health Malaysia (Ref: NMRR-21-763-59692).

#### 7.5.2. CO_2_ Data Acquisition

**Figure 10:**
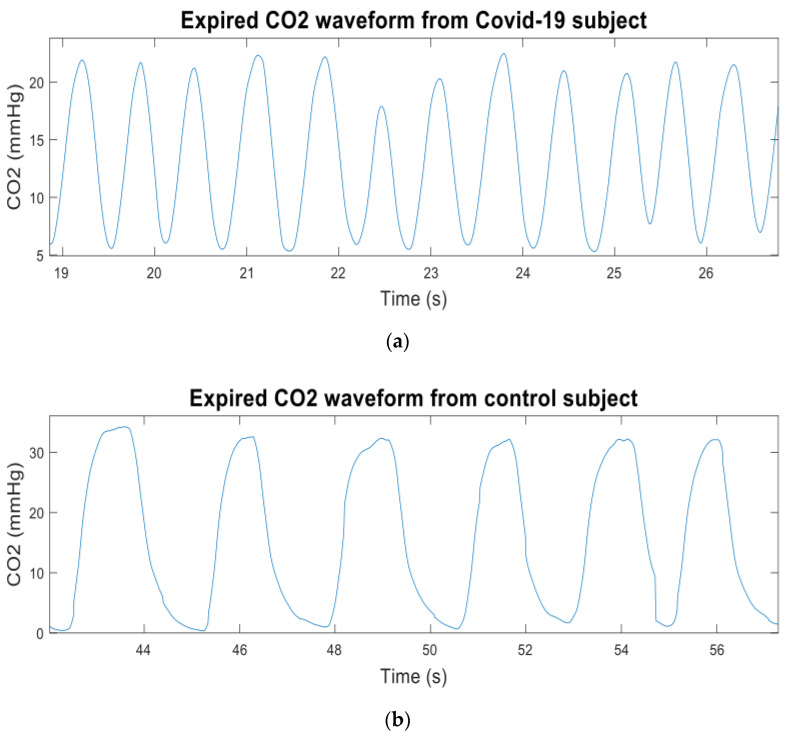
(**a**) Expired CO_2_ waveform from a Covid-19 subject; (**b**) expired CO_2_ waveform from a control subject.

The CO_2_ data were recorded using a newly developed sidestream CO_2_ measurement device based on human respiration, which can digitize the CO_2_ signal 100 times per second with 0.01-s intervals [90]. The data were derived from patients with COVID-19 confirmed using a PCR test. For comparison, CO_2_ data from control subjects were also recorded. The breath cycle was considered adequate when the CO_2_ waveform’s morphology was excellent and did not contain significant artifacts. “Artifact” refers to alternations in the morphology of CO_2_ waveforms induced by sneezing, talking, or coughing during data recording. Each patient breathed via the nasal cannula/sampling tube (Model 4000-7-25, Salter Labs, length—210 cm, internal diameter—1.27 mm, and prong diameter—1 mm). Figure 10a,b show the expired CO_2_ waveforms from the Covid-19 and the control subjects respectively. At least four valid breaths with regular morphology were used for further analysis from the CO_2_ signal of each participant recorded over approximately two minutes. Irregular and unstable CO_2_ waveforms were not considered for further investigation.

#### 7.5.3. Signal Analysis

**Figure 11:**
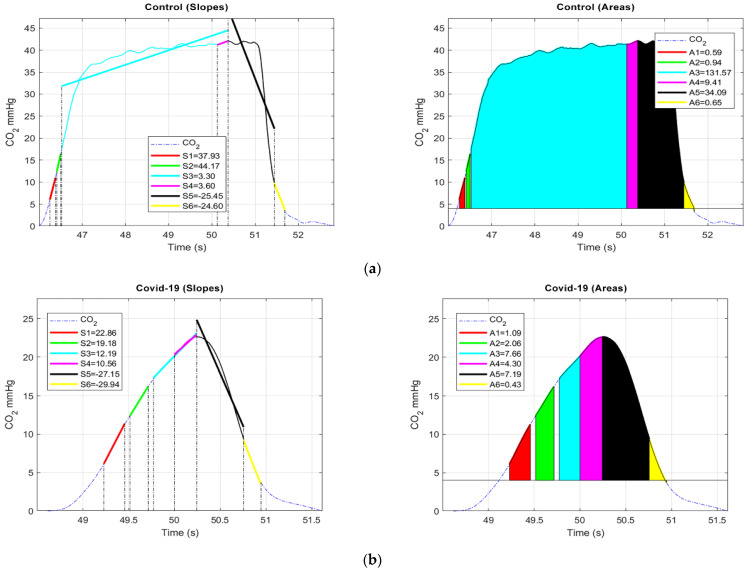
(**a**). Control: Left, illustration of a valid breath cycle from control patient with slope (*Sj*)*,* associated to six different regions mentioned above. Right, regions enclosed with colors represents the area (*AR_i_*) extracted from the CO_2_ signal associated to six regions mentioned; (**b**). COVID-19: Left, illustration of a valid breath cycle from COVID-19 patient slope (*Sj*) associated six regions mentioned above. Right, regions enclosed with colors represents the area (*AR_i_*) extracted from the CO_2_ signal associated to six regions mentioned.

Firstly, we decided to segment each valid set of four breath cycles into sub-cycles by employing the simple threshold method, as opposed to manual or visual inspection. Each cycle of four breaths was segmented into six regions by creating thresholds, as presented in Figure 11a,b. The threshold for each region was defined as follows:

(a) 1st sub-cycle: 6 mmHg (start) to 11 mmHg;
(b) 2nd sub-cycle: 12 mmHg to 16 mmHg;
(c) 3rd sub-cycle: 17 mmHg to EtCO_2_;
(d) 4th sub-cycle: 0.25 s from EtCO_2_ to EtCO_2_;
(e) 5th sub-cycle: EtCO_2_ to 10 mmHg;
(f) 6th sub-cycle: 10 mmHg to 4 mmHg (baseline).

Further, two features, area (*AR_i_*) and slope (*S_j_*), were computed from the segmented part of each breath cycle using Equations (1) and (2). The slopes of each sub-cycle were estimated using the general least squares linear fitting method. This computes the intercept and slope of the CO_2_ waveform by reducing the residue according to (2), which may permit the inclusion of the whole CO_2_ signal.

(1) $$AR_{i}=\frac{dt}{6}\overset{i}{\underset{j=0}{\sum }}(R_{j-1}\left(t\right)+4R_{j}\left(t\right)+R_{j+1}\left(t\right))$$

where *dt* and *R(t)* signify the sampling interval and CO_2_ signal, respectively.

(2) $$\mathrm{Slope}\;\left(S_{j}\right)=\frac{1}{C}\sum_{j=0}^{C-1}b_{j}{\left(M_{j}-s_{j}\right)}^{2}$$

where *C* is the length of slope (*S*), which reflects the CO_2_ signal, *b_j_* and *M_j_* are the *j*th element of weight and best linear fit, respectively, and *Sj* is the *j*th element of *S*. All of the features extracted and its significance are tabulated in Table 2 of the following section.

#### 7.5.4. Results

This preliminary study assessed the ability of CO_2_ waveform features to discriminate COVID-19 patients from a control group. In this preliminary report, we studied 14 patients, with 7 in each class. The significance of each feature extracted from the segmented regions of COVID-19 and control patients was identified using a paired *t*-test [102]. Statistical analysis was performed using SPSS (SPSS 23.0 for Windows) and the significance was set at *p* < 0.05.

Initially, the normality of each feature was verified based on skewness and kurtosis *z*-value, Shapiro–Wilk *p*-value, a Q–Q plot, and a histogram [103]. The results of the normality test show that the data were approximately normally distributed. Therefore, a parametric paired sample *t*-test was performed to verify the significance of the features, based on the *p*-values of all the sub-cycles.

Our findings suggest that the *area* and *slope* of sub-cycle 2 (12–16 mmHg) of the upward expiratory phase were slightly more significant (*p* < 0.05) than the alveolar and upper parts of the upward expiratory phase. We also noticed that the *slope (S_1_)* of the 6–11 mmHg region of the upward expiratory phase was significant (*p* = 0.003), while *S_5_* was insignificant (*p* = 0.01). From Table 1, it can be deduced that that the extracted features of *S_1_*, *S_2_*, *S_5_* and *AR_3_* exhibit acceptable discriminatory capabilities for the classes studied. This makes these features applicable for the screening and monitoring of respiratory illnesses, particularly COVID-19.

These findings, however, have several limitations. First, the useful information regarding SARS-CoV-2 is limited. Second, the information provided here is based on current data, but it may be altered as more data become available. We need to verify the applicability of these features with more samples and for different respiratory conditions, such as pneumonia, in order to confirm the deviation in these features for COVID-19. In the future, we must record CO_2_ data for a greater number of COVID-19 subjects, along with PEFR or spirometer data, to confirm the utility of capnographic indices and to determine the severity level of COVID-19 infection. Furthermore, adding more COVID data from all categories will facilitate a greater understanding of COVID-19. Hence, the viability of using the assessed features should be verified in future work.

## 8. Conclusions

An effective early testing device for SARS-CoV-2 infection will be of great use in reducing the spread of the virus. While vaccination programs are being used to reach herd immunity, many countries are still struggling to keep COVID-19 under control. Vaccination will not entirely prevent people from getting COVID-19. A fast, flexible and non-invasive testing device is urgently needed so that tests could be performed routinely and regularly. In light of this, this paper reviews and discusses the current methods available for assessing COVID-19. This review has several limitations. First, information regarding SARS-CoV-2 is limited. Second, the information provided here is based on the current evidence but may be modified as more information becomes available. Different studied have reported on the limitations of the currently available methods of diagnosing SARS-CoV-2 infection. Each testing technique has been shown to be suitable in different cases. Therefore, extensive research is still needed in order to develop alternative tools with enhanced accuracy in detecting SARS-CoV-2 infection at the early stage. In this preliminary study, we propose the use of CO_2_ patterns to screen for SARS-CoV-2 infection. This feature has been used to differentiate between respiratory conditions such as asthma, COPD and edema patients. It has proven the ability to classify asthmatic conditions based on capnogram features. In future studies, the feasibility of the same features will be verified for COVID-19. On the other hand, employing an amalgamation of capnogram features (e.g., the slope ratio, area ratio and frequency components) in the detection algorithm will provide greater understanding of COVID-19. Hence, the applicability of the studied features in a COVID-19 detection algorithm focusing on CO_2_ patterns should be verified in future work.

## Figures and Tables

**Table 1:** Different features extracted from the CO_2_ waveform and the types of classifiers applied.

Reference	Disease	Feature	Classifier	Performance Measure (Accuracy, Sensitivity, Specificity)/AUC	Limitations
You, B. et al. [84]	Asthma	S1, S2, S3, SR, A1 A2, SD1, SD2, SD3	-	*p* < 0.001 in all indices	Real-time implementation is still challenging due to the random time-based setting criteria
Hisamuddin et al. [87]	Asthma	Slope of phase 2, slope of phase 3, α angle	-	Angle α: *p* < 0.001 slope of phase 3: *p* < 0.001 slope of phase 2: *p* = 0.35	Selection bias of waveform for analysis
Kean et al. [88]	Asthma	Area (A1) and (A2), area ratio (AR), S1 and S2 (Slope), SR (slope ratio), α angle, HP1 and HP2 (activity), HP1 and HP2 (mobility), HP1 and HP2 (complexity)	-	*p* < 0.0001 (SR) *p* = 0.0001051 (HP2 mobility)	Capnogram features were extracted manually
Betancourt et al. [86]	Asthma	Wavelet coefficients	Support vector machine	sensitivity: 55.71%, specificity: 99.38%,	Improper prediction of asthma severity degree 1
Doğan, Nurettin Özgür et al. [89]	COPD	EtCO_2_	-	sensitivity: 65.2% specificity: 63.6%	Small sample size The mean bias of the study was 4.68 ± 7.21
Mieloszyk et al. [83]	COPD, CHF, normal subject	Exhalation duration, Pet CO_2,_ time spent at Pet CO_2_, exhalation slope	Quadratic discriminant analysis	Accuracy: 93.9%, for COPD/normal classification Accuracy: 80.0%, for COPD/CHF classification	Inability of tracking changes in disease severity and response to treatment over time Some patients presented with a mixed picture of CHF and COPD
Herry, C. L et al. [90]	Breath classification (normal or abnormal) in intubated patients in ICU	Plateau slope, residuals, TO angle, α angle, β angle, PeakCO_2_, SR1, min plateau, skew, kurtosis, inspiration slope, expiration slope, width, sharpness, MinCO_2_	Decision tree (DT), k-nearest neighbors (KNN), and naive Bayes (NB)	AUC: 90% (DT) AUC: 89%(KNN) AUC: 88%(NB)	The type of abnormalities was not classified
Singh, O. P., Palaniappan, R., and Malarvili, M. B. [91]	Asthma	Upward expiration (AR1), downward inspiration (AR2), and the sum of AR1 and AR2	Support vector machine (SVM) k-nearest neighbor (k-NN) and naive Bayes (NB)	Average accuracy of 94.52%, sensitivity of 97.67%, and specificity of 90%	-
El-Badawy, I. M., Singh, O. P., and Omar, Z. [85]	Differentiation of regular and irregular capnograms	Energy, variance, skewness and kurtosis, number of relatively high spectral peaks and the area under the normalized magnitude spectrum	Support vector machine	accuracy: 86.5% specificity: 84% sensitivity: 89% precision: 86.51%	On average, 13.5% of the capnogram segments were misclassified due to the overlap between some regular and irregular capnogram samples

**Table 2:** Features and P-values for different segmented sub-cycles extracted from the CO_2_ waveform.

S. No.	Segmented Sub-Cycles	Features	*p*-Value
1	6–11 mmHg	*A_1_*	0.05
		*S_1_*	0.003
2	12–16 mmHg	*A_2_*	0.01
		*S_2_*	0.002
3	17 mmHg–EtCO_2_	*A_3_*	0.001
		*S_3_*	0.08
4	0.25 s from EtCO_2_ to EtCO_2_	*A_4_*	0.07
		*S_4_*	0.05
5	EtCO_2_–10 mmHg	*A_5_*	0.07
		*S_5_*	0.01
6	10 mmHg–4 mmHg	*A_6_*	0.09
		*S_6_*	0.08

## Data Availability

The data presented in this study are available on request from the corresponding author. The data are not publicly available at the moment because the data collection is not completed and still at preliminary stage.
